# [(1R*,2S*)-*N* ^1^-Benzyl-2-phenyl-1-(pyridin-2-yl)-*N* ^2^-(pyridin-2-ylmeth­yl)ethane-1,2-diamine]­dichloridozinc(II)

**DOI:** 10.1107/S1600536811004314

**Published:** 2011-02-16

**Authors:** Adailton J. Bortoluzzi, Sandro L. Mireski, Antonio C. Joussef

**Affiliations:** a Depto. de Química – UFSC, 88040-900 Florianópolis, SC, Brazil

## Abstract

In the mononuclear zinc title complex, [ZnCl_2_(C_26_H_26_N_4_)], the Zn^II^ ion is surrounded by three N atoms from a (1R*,2S*)-*N* ^1^-benzyl-2-phenyl-1-(pyridin-2-yl)-*N* ^2^-(pyridin-2-ylmeth­yl)ethane-1,2-diamine (BPPPEN) ligand and two terminal chloride ligands, resulting in a highly distorted environment around the metal atom. The calculated τ parameter of 0.42 indicates that the coordination geometry is approximately square-pyramidal. Hydrogen bonds involving centrosymmetric N—H⋯Cl inter­actions form dimeric structures. The mol­ecules are stacked along the *a* and *b* axes.

## Related literature

For general background to the chemistry and biological properties of vicinal diamines, see: Bennani & Hanessian (1997 ▶); Lucet *et al.* (1998 ▶); Fache *et al.* (2000 ▶); Saibabu  Kotti*et al.* (2006) ▶ Alexakis & Andrey (2002 ▶); Andrey *et al.* (2003 ▶); Ma *et al.* (2003 ▶); Notz *et al.* (2004 ▶); Bassindale *et al.* (2004 ▶); Mealy *et al.* (2004 ▶). For a related structure, see: Mikata *et al.* (2009 ▶). For coordination geom­etries, see: Addison *et al.* (1984 ▶). For hydrogen bonds, see: Steiner (2002 ▶).
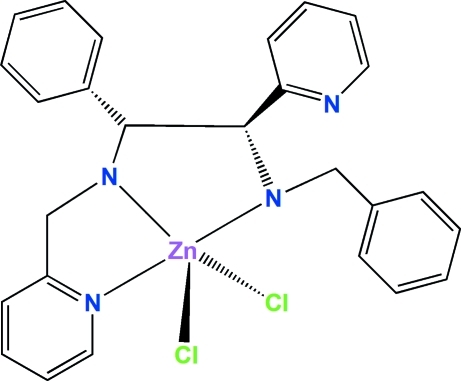


## Experimental

### 

#### Crystal data

[ZnCl_2_(C_26_H_26_N_4_)]
*M* *_r_* = 530.78
Monoclinic, 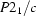

*a* = 9.1716 (14) Å
*b* = 28.888 (2) Å
*c* = 10.4304 (12) Å
β = 109.541 (8)°
*V* = 2604.3 (5) Å^3^
*Z* = 4
Mo *K*α radiation
μ = 1.17 mm^−1^
*T* = 293 K
0.46 × 0.43 × 0.26 mm

#### Data collection

Enraf–Nonius CAD-4 diffractometer
Absorption correction: ψ scan [*PLATON* (Spek, 2009 ▶); North *et al.* (1968 ▶)] *T*_min_ = 0.615, *T*_max_ = 0.751
4899 measured reflections
4632 independent reflections
2892 reflections with *I* > 2σ(*I*)
*R*_int_ = 0.048
3 standard reflections every 200 reflections  intensity decay: 1%

#### Refinement

*R*[*F* ^2^ > 2σ(*F* ^2^)] = 0.056
*wR*(*F* ^2^) = 0.169
*S* = 1.05
4632 reflections
298 parameters
H-atom parameters constrained
Δρ_max_ = 0.74 e Å^−3^
Δρ_min_ = −0.39 e Å^−3^

### 

Data collection: *CAD-4 Software* (Enraf–Nonius, 1989 ▶); cell refinement: *SET4* in *CAD-4 Software*; data reduction: *HELENA* (Spek, 1996 ▶); program(s) used to solve structure: *SIR97* (Altomare *et al.*, 1999 ▶); program(s) used to refine structure: *SHELXL97* (Sheldrick, 2008 ▶); molecular graphics: *PLATON* (Spek, 2009 ▶) and *Mercury* (Macrae *et al.*, 2006 ▶); software used to prepare material for publication: *SHELXL97*.

## Supplementary Material

Crystal structure: contains datablocks global, I. DOI: 10.1107/S1600536811004314/zj2001sup1.cif

Structure factors: contains datablocks I. DOI: 10.1107/S1600536811004314/zj2001Isup2.hkl

Additional supplementary materials:  crystallographic information; 3D view; checkCIF report

## Figures and Tables

**Table 1:** Hydrogen-bond geometry (Å, °)

*D*—H⋯*A*	*D*—H	H⋯*A*	*D*⋯*A*	*D*—H⋯*A*
N4—H4⋯Cl2^i^	0.91	2.43	3.304 (5)	160

Symmetry code: (i) 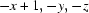.

**Fig. 1.**
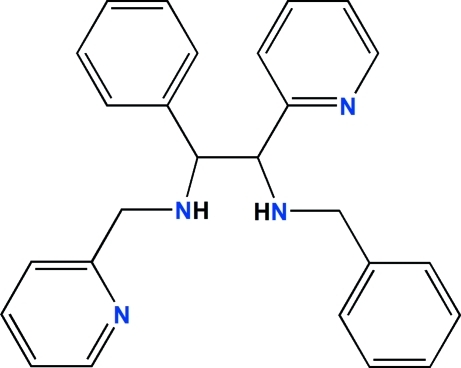
(1R*, 2S*)-N1-benzyl-2-phenyl-1-(pyridin-2-yl)-N2-[(pyridin-2-yl) methyl] ethane-1, 2-diamine

**Fig. 2.**
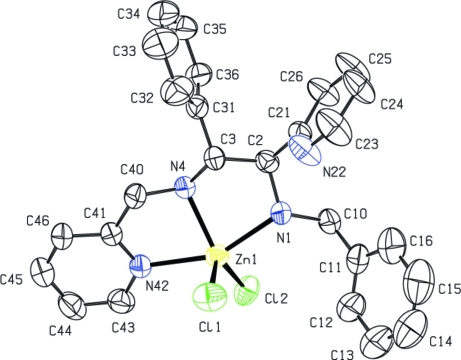
The molecular structure of complex (I) showing the atom-labelling scheme. Ellipsoids are drawn at the 50% probability level. Hydrogen atoms were omitted for clarity.

**Fig. 3.**
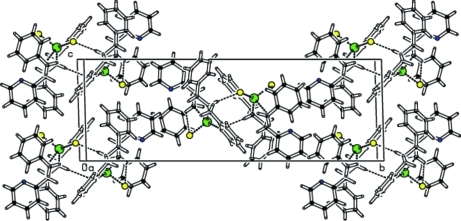
Packing of the title compound with hydrogen bonding.

## supplementary crystallographic information

### Comment

There has been much interest in developing methods for making vicinal diamines and their derivatives since they are important in medicinal chemistry, natural products, coordination chemistry and asymmetric catalysis (Bennani & Hanessian, 1997; Lucet *et al.*, 1998; Fache *et al.*, 2000; Saibabu Kotti *et al.*, 2006). They have been extensively used as ligands and catalysts in synthesis with impressive results (Alexakis & Andrey, 2002; Andrey *et al.*, 2003; Ma *et al.*, 2003; Notz *et al.*, 2004; Bassindale *et al.*, 2004; Mealy *et al.*, 2004). We report here the crystal structure of the title new zinc complex with the chelating diamine (1*R**, 2S*)-N1-benzyl-2-phenyl-1-(pyridin-2-yl)-N2-[(pyridin-2-yl) methyl] ethane-1, 2-diamine (Fig. 1). In our study of vicinal diamines, we isolated the title complex from a mixture of stereo isomeric diamines in the presence of anhydrous zinc chloride in methanol.

Fig. 2 shows the molecular structure of thr title compound. It is a neutral mononuclear zinc complex, where Zn^II^ ion is surrounded by three nitrogen atoms from BPPPEN ligand and two chloro terminal ligands, resulting in a highly distorted environment around the metal center. The calculated τ parameter of 0.42 indicate the coordination geometry has lightly square pyramidal character (Addison *et al.*, 1984).

Centrosymmetric hydrogen bonds form dimmeric structures through N4—H4··· Cl2 interactions (Fig. 3). The geometric parameters of these interactions are in agreement with those postulated by Steiner (2002). The packing analysis shows that the molecules are stacked along a and b crystallographic axes.

### Experimental

To a solution of BPPPEN (0,5 g; 1,24 mmol) in methanol (25 mL) was added anhydrous ZnCl2 (0,173 g; 1,27 mmol) and the mixture was heated at reflux until all zinc chloride dissolved. The solution was allowed to cool slowly at room temperature and a white crystalline solid, suitable for X-ray crystallographic analysis, was collected after one week (0,177 g; 26%).

### Refinement

H atoms were placed at their idealized positions with distances of 0.93, 0.98 and 0.97 Å and *U*_eq_ fixed at 1.2 times *U*_iso_ of the preceding atom for CH_Ar_, CH and CH_2_, respectively. Hydrogen atoms of the amine groups were found from Fourier difference map and treated with riding model.

### Crystal data

**Table:**

[ZnCl_2_(C_26_H_26_N_4_)]	*F*(000) = 1096
*M**_r_* = 530.78	*D*_x_ = 1.354 Mg m^−^^3^
Monoclinic, *P*2_1_/*c*	Mo *K*α radiation, λ = 0.71069 Å
Hall symbol: -P 2ybc	Cell parameters from 25 reflections
*a* = 9.1716 (14) Å	θ = 8.4–13.9°
*b* = 28.888 (2) Å	µ = 1.17 mm^−^^1^
*c* = 10.4304 (12) Å	*T* = 293 K
β = 109.541 (8)°	Disc, colorless
*V* = 2604.3 (5) Å^3^	0.46 × 0.43 × 0.26 mm
*Z* = 4	

### Data collection

**Table:**

Enraf–Nonius CAD-4 diffractometer	2892 reflections with *I* > 2σ(*I*)
Radiation source: fine-focus sealed tube	*R*_int_ = 0.048
graphite	θ_max_ = 25.1°, θ_min_ = 1.4°
ω–2θ scans	*h* = −10→10
Absorption correction: ψ scan [*PLATON* (Spek, 2009); North *et al.* (1968)]	*k* = −34→0
*T*_min_ = 0.615, *T*_max_ = 0.751	*l* = −12→0
4899 measured reflections	3 standard reflections every 200 reflections
4632 independent reflections	intensity decay: 1%

### Refinement

**Table:**

Refinement on *F*^2^	Primary atom site location: structure-invariant direct methods
Least-squares matrix: full	Secondary atom site location: difference Fourier map
*R*[*F*^2^ > 2σ(*F*^2^)] = 0.056	Hydrogen site location: inferred from neighbouring sites
*wR*(*F*^2^) = 0.169	H-atom parameters constrained
*S* = 1.05	*w* = 1/[σ^2^(*F*_o_^2^) + (0.0767*P*)^2^ + 4.3127*P*] where *P* = (*F*_o_^2^ + 2*F*_c_^2^)/3
4632 reflections	(Δ/σ)_max_ < 0.001
298 parameters	Δρ_max_ = 0.74 e Å^−^^3^
0 restraints	Δρ_min_ = −0.39 e Å^−^^3^

### Fractional atomic coordinates and isotropic or equivalent isotropic displacement parameters (Å^2^)

**Table:**

	*x*	*y*	*z*	*U*_iso_*/*U*_eq_	
Zn1	0.46704 (7)	0.07907 (2)	−0.11615 (6)	0.0407 (2)	
Cl1	0.4754 (2)	0.13915 (6)	−0.24916 (18)	0.0689 (5)	
Cl2	0.26694 (16)	0.02852 (5)	−0.17505 (17)	0.0541 (4)	
N1	0.4102 (5)	0.12227 (17)	0.0387 (4)	0.0422 (11)	
H1	0.4033	0.1480	−0.0152	0.051*	
C2	0.5396 (6)	0.1186 (2)	0.1695 (5)	0.0423 (13)	
H2	0.5278	0.0898	0.2146	0.051*	
C3	0.6918 (6)	0.11640 (18)	0.1384 (5)	0.0376 (12)	
H3	0.6986	0.1447	0.0888	0.045*	
N4	0.6810 (5)	0.07700 (17)	0.0455 (4)	0.0412 (10)	
H4	0.6723	0.0492	0.0841	0.049*	
C10	0.2536 (7)	0.1176 (2)	0.0553 (6)	0.0527 (16)	
H10A	0.2160	0.0863	0.0328	0.063*	
H10B	0.2636	0.1233	0.1496	0.063*	
C11	0.1394 (7)	0.1508 (2)	−0.0336 (7)	0.0578 (17)	
C12	0.0595 (8)	0.1409 (3)	−0.1672 (8)	0.073 (2)	
H12	0.0785	0.1129	−0.2027	0.088*	
C13	−0.0465 (11)	0.1704 (4)	−0.2503 (10)	0.107 (3)	
H13	−0.1007	0.1622	−0.3398	0.128*	
C14	−0.0720 (13)	0.2124 (4)	−0.1994 (14)	0.126 (4)	
H14	−0.1410	0.2332	−0.2562	0.151*	
C15	0.0021 (14)	0.2238 (4)	−0.0674 (14)	0.131 (4)	
H15	−0.0167	0.2521	−0.0332	0.158*	
C16	0.1102 (10)	0.1915 (3)	0.0184 (10)	0.097 (3)	
H16	0.1604	0.1985	0.1097	0.116*	
C21	0.5408 (6)	0.1586 (2)	0.2628 (5)	0.0439 (14)	
N22	0.5383 (8)	0.1999 (2)	0.2084 (6)	0.0710 (17)	
C23	0.5386 (11)	0.2370 (3)	0.2849 (8)	0.089 (3)	
H23	0.5390	0.2661	0.2473	0.106*	
C24	0.5384 (11)	0.2342 (3)	0.4158 (8)	0.085 (2)	
H24	0.5347	0.2607	0.4655	0.102*	
C25	0.5437 (11)	0.1911 (3)	0.4705 (8)	0.086 (2)	
H25	0.5468	0.1876	0.5600	0.103*	
C26	0.5446 (9)	0.1531 (3)	0.3938 (6)	0.0666 (19)	
H26	0.5478	0.1236	0.4303	0.080*	
C31	0.8333 (6)	0.1148 (2)	0.2652 (6)	0.0462 (14)	
C32	0.9515 (8)	0.1457 (3)	0.2857 (8)	0.081 (2)	
H32	0.9441	0.1686	0.2211	0.097*	
C33	1.0832 (10)	0.1437 (4)	0.4016 (10)	0.103 (3)	
H33	1.1622	0.1652	0.4146	0.124*	
C34	1.0948 (10)	0.1104 (4)	0.4943 (9)	0.094 (3)	
H34	1.1827	0.1093	0.5715	0.112*	
C35	0.9809 (9)	0.0781 (3)	0.4783 (7)	0.082 (2)	
H35	0.9905	0.0553	0.5436	0.099*	
C36	0.8510 (7)	0.0802 (2)	0.3625 (6)	0.0588 (16)	
H36	0.7738	0.0581	0.3493	0.071*	
C40	0.8042 (6)	0.0759 (2)	−0.0161 (6)	0.0516 (15)	
H40A	0.9018	0.0676	0.0525	0.062*	
H40B	0.8154	0.1063	−0.0511	0.062*	
C41	0.7654 (6)	0.0413 (2)	−0.1293 (6)	0.0451 (14)	
N42	0.6161 (5)	0.03297 (17)	−0.1926 (5)	0.0456 (12)	
C43	0.5756 (8)	0.0035 (2)	−0.2951 (6)	0.0565 (16)	
H43	0.4708	−0.0019	−0.3392	0.068*	
C44	0.6810 (9)	−0.0196 (3)	−0.3402 (7)	0.068 (2)	
H44	0.6488	−0.0404	−0.4124	0.082*	
C45	0.8357 (9)	−0.0108 (3)	−0.2740 (7)	0.070 (2)	
H45	0.9104	−0.0257	−0.3011	0.084*	
C46	0.8785 (7)	0.0199 (2)	−0.1688 (7)	0.0590 (17)	
H46	0.9825	0.0263	−0.1241	0.071*	

### Atomic displacement parameters (Å^2^)

**Table:**

	*U*^11^	*U*^22^	*U*^33^	*U*^12^	*U*^13^	*U*^23^
Zn1	0.0376 (3)	0.0438 (4)	0.0395 (3)	−0.0025 (3)	0.0115 (2)	0.0016 (3)
Cl1	0.0804 (12)	0.0628 (11)	0.0625 (10)	−0.0105 (9)	0.0225 (9)	0.0193 (9)
Cl2	0.0394 (8)	0.0452 (9)	0.0764 (11)	−0.0050 (6)	0.0176 (7)	−0.0015 (8)
N1	0.038 (3)	0.051 (3)	0.041 (2)	0.004 (2)	0.017 (2)	0.001 (2)
C2	0.049 (3)	0.042 (3)	0.038 (3)	0.001 (3)	0.017 (3)	0.004 (2)
C3	0.039 (3)	0.034 (3)	0.043 (3)	−0.003 (2)	0.019 (2)	0.000 (2)
N4	0.037 (2)	0.050 (3)	0.038 (2)	0.001 (2)	0.0144 (19)	−0.002 (2)
C10	0.046 (3)	0.071 (4)	0.047 (3)	−0.001 (3)	0.023 (3)	−0.003 (3)
C11	0.048 (4)	0.061 (4)	0.072 (4)	0.008 (3)	0.029 (3)	0.006 (4)
C12	0.068 (5)	0.074 (5)	0.073 (5)	0.018 (4)	0.017 (4)	0.006 (4)
C13	0.091 (7)	0.120 (9)	0.097 (7)	0.040 (6)	0.014 (5)	0.010 (6)
C14	0.107 (8)	0.129 (10)	0.133 (10)	0.062 (7)	0.028 (7)	0.021 (8)
C15	0.131 (10)	0.107 (9)	0.165 (12)	0.057 (8)	0.062 (9)	−0.009 (8)
C16	0.091 (6)	0.099 (7)	0.105 (7)	0.026 (5)	0.040 (5)	−0.012 (6)
C21	0.049 (3)	0.048 (4)	0.039 (3)	0.003 (3)	0.020 (3)	0.008 (3)
N22	0.121 (5)	0.050 (4)	0.055 (3)	0.002 (3)	0.047 (4)	−0.001 (3)
C23	0.162 (9)	0.037 (4)	0.087 (6)	0.003 (5)	0.068 (6)	−0.005 (4)
C24	0.135 (7)	0.067 (5)	0.070 (5)	0.004 (5)	0.057 (5)	−0.026 (4)
C25	0.137 (7)	0.083 (6)	0.056 (4)	−0.012 (5)	0.059 (5)	−0.007 (4)
C26	0.106 (6)	0.055 (4)	0.048 (4)	−0.002 (4)	0.037 (4)	0.000 (3)
C31	0.043 (3)	0.048 (4)	0.048 (3)	0.000 (3)	0.015 (3)	−0.014 (3)
C32	0.063 (5)	0.092 (6)	0.078 (5)	−0.020 (4)	0.013 (4)	−0.011 (4)
C33	0.069 (6)	0.134 (9)	0.092 (7)	−0.028 (6)	0.008 (5)	−0.036 (7)
C34	0.067 (6)	0.133 (9)	0.063 (5)	0.011 (6)	−0.002 (4)	−0.032 (6)
C35	0.076 (5)	0.108 (7)	0.050 (4)	0.023 (5)	0.004 (4)	−0.017 (4)
C36	0.054 (4)	0.067 (4)	0.048 (3)	0.011 (3)	0.006 (3)	−0.008 (4)
C40	0.037 (3)	0.065 (4)	0.053 (3)	0.000 (3)	0.017 (3)	−0.006 (3)
C41	0.042 (3)	0.054 (4)	0.042 (3)	0.002 (3)	0.017 (3)	−0.004 (3)
N42	0.039 (3)	0.056 (3)	0.043 (3)	0.000 (2)	0.015 (2)	−0.006 (2)
C43	0.055 (4)	0.065 (4)	0.047 (3)	−0.007 (3)	0.015 (3)	−0.006 (3)
C44	0.082 (5)	0.073 (5)	0.055 (4)	0.002 (4)	0.030 (4)	−0.013 (4)
C45	0.067 (5)	0.088 (6)	0.063 (4)	0.014 (4)	0.031 (4)	−0.010 (4)
C46	0.043 (3)	0.081 (5)	0.056 (4)	0.007 (3)	0.022 (3)	−0.006 (4)

### Geometric parameters (Å, °)

**Table:**

Zn1—N4	2.118 (4)	N22—C23	1.334 (9)
Zn1—N1	2.236 (4)	C23—C24	1.369 (10)
Zn1—N42	2.238 (5)	C23—H23	0.9300
Zn1—Cl1	2.2393 (17)	C24—C25	1.366 (11)
Zn1—Cl2	2.2635 (16)	C24—H24	0.9300
N1—C2	1.482 (7)	C25—C26	1.359 (10)
N1—C10	1.509 (7)	C25—H25	0.9300
N1—H1	0.9223	C26—H26	0.9300
C2—C21	1.509 (8)	C31—C32	1.364 (9)
C2—C3	1.537 (7)	C31—C36	1.395 (9)
C2—H2	0.9800	C32—C33	1.396 (11)
C3—N4	1.476 (7)	C32—H32	0.9300
C3—C31	1.513 (8)	C33—C34	1.342 (13)
C3—H3	0.9800	C33—H33	0.9300
N4—C40	1.476 (7)	C34—C35	1.369 (12)
N4—H4	0.9146	C34—H34	0.9300
C10—C11	1.492 (9)	C35—C36	1.386 (9)
C10—H10A	0.9700	C35—H35	0.9300
C10—H10B	0.9700	C36—H36	0.9300
C11—C16	1.358 (10)	C40—C41	1.496 (8)
C11—C12	1.371 (10)	C40—H40A	0.9700
C12—C13	1.362 (11)	C40—H40B	0.9700
C12—H12	0.9300	C41—N42	1.328 (7)
C13—C14	1.375 (14)	C41—C46	1.384 (8)
C13—H13	0.9300	N42—C43	1.318 (8)
C14—C15	1.356 (15)	C43—C44	1.381 (9)
C14—H14	0.9300	C43—H43	0.9300
C15—C16	1.436 (13)	C44—C45	1.378 (10)
C15—H15	0.9300	C44—H44	0.9300
C16—H16	0.9300	C45—C46	1.362 (9)
C21—N22	1.318 (8)	C45—H45	0.9300
C21—C26	1.365 (8)	C46—H46	0.9300
			
N4—Zn1—N1	79.54 (16)	N22—C21—C26	121.9 (6)
N4—Zn1—N42	75.71 (17)	N22—C21—C2	114.8 (5)
N1—Zn1—N42	155.13 (16)	C26—C21—C2	123.3 (5)
N4—Zn1—Cl1	107.49 (14)	C21—N22—C23	118.2 (6)
N1—Zn1—Cl1	94.56 (14)	N22—C23—C24	123.4 (7)
N42—Zn1—Cl1	95.09 (14)	N22—C23—H23	118.3
N4—Zn1—Cl2	130.48 (14)	C24—C23—H23	118.3
N1—Zn1—Cl2	101.09 (12)	C25—C24—C23	117.2 (7)
N42—Zn1—Cl2	93.20 (13)	C25—C24—H24	121.4
Cl1—Zn1—Cl2	121.64 (7)	C23—C24—H24	121.4
C2—N1—C10	112.8 (4)	C26—C25—C24	119.8 (7)
C2—N1—Zn1	108.5 (3)	C26—C25—H25	120.1
C10—N1—Zn1	119.6 (3)	C24—C25—H25	120.1
C2—N1—H1	119.3	C25—C26—C21	119.5 (7)
C10—N1—H1	105.6	C25—C26—H26	120.3
Zn1—N1—H1	89.7	C21—C26—H26	120.3
N1—C2—C21	111.7 (4)	C32—C31—C36	117.5 (6)
N1—C2—C3	108.2 (4)	C32—C31—C3	121.5 (6)
C21—C2—C3	110.9 (5)	C36—C31—C3	121.0 (5)
N1—C2—H2	108.6	C31—C32—C33	121.4 (9)
C21—C2—H2	108.6	C31—C32—H32	119.3
C3—C2—H2	108.6	C33—C32—H32	119.3
N4—C3—C31	113.6 (4)	C34—C33—C32	119.4 (9)
N4—C3—C2	107.7 (4)	C34—C33—H33	120.3
C31—C3—C2	113.0 (4)	C32—C33—H33	120.3
N4—C3—H3	107.4	C33—C34—C35	121.9 (8)
C31—C3—H3	107.4	C33—C34—H34	119.1
C2—C3—H3	107.4	C35—C34—H34	119.1
C3—N4—C40	114.2 (4)	C34—C35—C36	118.3 (8)
C3—N4—Zn1	110.0 (3)	C34—C35—H35	120.9
C40—N4—Zn1	107.1 (3)	C36—C35—H35	120.9
C3—N4—H4	112.7	C35—C36—C31	121.5 (7)
C40—N4—H4	111.4	C35—C36—H36	119.2
Zn1—N4—H4	100.4	C31—C36—H36	119.2
C11—C10—N1	111.7 (5)	N4—C40—C41	110.1 (5)
C11—C10—H10A	109.3	N4—C40—H40A	109.6
N1—C10—H10A	109.3	C41—C40—H40A	109.6
C11—C10—H10B	109.3	N4—C40—H40B	109.6
N1—C10—H10B	109.3	C41—C40—H40B	109.6
H10A—C10—H10B	108.0	H40A—C40—H40B	108.2
C16—C11—C12	118.5 (7)	N42—C41—C46	121.5 (6)
C16—C11—C10	119.9 (7)	N42—C41—C40	116.5 (5)
C12—C11—C10	121.6 (6)	C46—C41—C40	122.0 (5)
C13—C12—C11	122.8 (8)	C43—N42—C41	118.9 (5)
C13—C12—H12	118.6	C43—N42—Zn1	129.3 (4)
C11—C12—H12	118.6	C41—N42—Zn1	111.5 (4)
C12—C13—C14	118.9 (10)	N42—C43—C44	123.3 (6)
C12—C13—H13	120.6	N42—C43—H43	118.3
C14—C13—H13	120.6	C44—C43—H43	118.3
C15—C14—C13	121.0 (10)	C45—C44—C43	117.4 (6)
C15—C14—H14	119.5	C45—C44—H44	121.3
C13—C14—H14	119.5	C43—C44—H44	121.3
C14—C15—C16	118.8 (10)	C46—C45—C44	119.7 (6)
C14—C15—H15	120.6	C46—C45—H45	120.2
C16—C15—H15	120.6	C44—C45—H45	120.2
C11—C16—C15	120.0 (9)	C45—C46—C41	119.2 (6)
C11—C16—H16	120.0	C45—C46—H46	120.4
C15—C16—H16	120.0	C41—C46—H46	120.4
			
N4—Zn1—N1—C2	−9.2 (3)	C26—C21—N22—C23	−0.4 (11)
N42—Zn1—N1—C2	−3.6 (6)	C2—C21—N22—C23	179.7 (7)
Cl1—Zn1—N1—C2	−116.2 (3)	C21—N22—C23—C24	−1.3 (14)
Cl2—Zn1—N1—C2	120.3 (3)	N22—C23—C24—C25	2.4 (15)
N4—Zn1—N1—C10	−140.5 (4)	C23—C24—C25—C26	−1.9 (14)
N42—Zn1—N1—C10	−134.9 (4)	C24—C25—C26—C21	0.3 (13)
Cl1—Zn1—N1—C10	112.5 (4)	N22—C21—C26—C25	0.9 (11)
Cl2—Zn1—N1—C10	−11.0 (4)	C2—C21—C26—C25	−179.3 (7)
C10—N1—C2—C21	−66.1 (6)	N4—C3—C31—C32	110.5 (7)
Zn1—N1—C2—C21	159.1 (4)	C2—C3—C31—C32	−126.4 (6)
C10—N1—C2—C3	171.6 (5)	N4—C3—C31—C36	−66.3 (7)
Zn1—N1—C2—C3	36.7 (5)	C2—C3—C31—C36	56.8 (7)
N1—C2—C3—N4	−56.4 (5)	C36—C31—C32—C33	−1.9 (11)
C21—C2—C3—N4	−179.2 (4)	C3—C31—C32—C33	−178.8 (7)
N1—C2—C3—C31	177.3 (4)	C31—C32—C33—C34	0.7 (14)
C21—C2—C3—C31	54.5 (6)	C32—C33—C34—C35	0.2 (14)
C31—C3—N4—C40	−65.8 (6)	C33—C34—C35—C36	0.2 (13)
C2—C3—N4—C40	168.2 (4)	C34—C35—C36—C31	−1.5 (10)
C31—C3—N4—Zn1	173.7 (4)	C32—C31—C36—C35	2.3 (9)
C2—C3—N4—Zn1	47.7 (5)	C3—C31—C36—C35	179.2 (6)
N1—Zn1—N4—C3	−21.3 (3)	C3—N4—C40—C41	−168.0 (5)
N42—Zn1—N4—C3	161.2 (4)	Zn1—N4—C40—C41	−46.0 (6)
Cl1—Zn1—N4—C3	70.3 (3)	N4—C40—C41—N42	27.5 (8)
Cl2—Zn1—N4—C3	−117.0 (3)	N4—C40—C41—C46	−154.3 (6)
N1—Zn1—N4—C40	−145.9 (4)	C46—C41—N42—C43	0.2 (9)
N42—Zn1—N4—C40	36.5 (4)	C40—C41—N42—C43	178.5 (6)
Cl1—Zn1—N4—C40	−54.4 (4)	C46—C41—N42—Zn1	−173.9 (5)
Cl2—Zn1—N4—C40	118.4 (4)	C40—C41—N42—Zn1	4.4 (7)
C2—N1—C10—C11	139.8 (5)	N4—Zn1—N42—C43	163.2 (6)
Zn1—N1—C10—C11	−90.9 (6)	N1—Zn1—N42—C43	157.5 (5)
N1—C10—C11—C16	−97.6 (7)	Cl1—Zn1—N42—C43	−90.0 (5)
N1—C10—C11—C12	83.7 (8)	Cl2—Zn1—N42—C43	32.2 (5)
C16—C11—C12—C13	0.7 (13)	N4—Zn1—N42—C41	−23.4 (4)
C10—C11—C12—C13	179.3 (8)	N1—Zn1—N42—C41	−29.1 (7)
C11—C12—C13—C14	1.7 (15)	Cl1—Zn1—N42—C41	83.3 (4)
C12—C13—C14—C15	−2.6 (18)	Cl2—Zn1—N42—C41	−154.5 (4)
C13—C14—C15—C16	1(2)	C41—N42—C43—C44	0.4 (10)
C12—C11—C16—C15	−2.3 (13)	Zn1—N42—C43—C44	173.3 (5)
C10—C11—C16—C15	179.0 (8)	N42—C43—C44—C45	−0.5 (11)
C14—C15—C16—C11	1.5 (17)	C43—C44—C45—C46	−0.1 (11)
N1—C2—C21—N22	−52.8 (7)	C44—C45—C46—C41	0.7 (11)
C3—C2—C21—N22	68.1 (7)	N42—C41—C46—C45	−0.8 (10)
N1—C2—C21—C26	127.3 (6)	C40—C41—C46—C45	−179.0 (6)
C3—C2—C21—C26	−111.8 (7)		

### Hydrogen-bond geometry (Å, °)

**Table:**

*D*—H···*A*	*D*—H	H···*A*	*D*···*A*	*D*—H···*A*
N4—H4···Cl2^i^	0.91	2.43	3.304 (5)	160

Symmetry codes: (i) −*x*+1, −*y*, −*z*.
